# Circulating lipids and breast cancer prognosis in the Malmö diet and cancer study

**DOI:** 10.1007/s10549-021-06462-7

**Published:** 2021-11-25

**Authors:** Sixten Harborg, Thomas P. Ahern, Maria Feldt, Ann H. Rosendahl, Deirdre Cronin-Fenton, Olle Melander, Signe Borgquist

**Affiliations:** 1 Department of Oncology, Aarhus University Hospital/Aarhus University, Entrance C, Level 1, C118, Palle Juul-Jensens Boulevard 99, 8200 Aarhus N, Denmark; 2 Department of Clinical Medicine and Department of Clinical Epidemiology, Aarhus University, Aarhus, Denmark; 3 Department of Surgery, Larner College of Medicine, University of Vermont, Burlington, USA; 4 Department of Clinical Sciences Lund, Oncology, Skåne University Hospital, Lund University, Lund, Sweden; 5 Department of Clinical Sciences Malmö, Hypertension and Cardiovascular Disease, Lund University, Malmö, Sweden; 6 Department of Clinical Sciences Malmö, Lund University Diabetes Centre, Lund University Malmö, Malmö, Sweden; 7 Clinical Research Centre, Skåne University Hospital, Lund and Malmö, Malmö, Sweden; 8 Department of Emergency and Internal Medicine, Skåne University Hospital, Malmö, Sweden; 9 Department of Cardiology, Skåne University Hospital, Malmö, Sweden

**Keywords:** Cancer, Breast cancer, Circulating lipids, Dyslipidemia, Lipids, Apolipoproteins, Apolipoprotein A1, Apolipoprotein B, Prognosis, Recurrence, Loco-regional recurrence, Distant recurrence, Survival, All-cause mortality, Cohort study

## Abstract

**Purpose:**

Examine the association between circulating lipids and breast cancer outcomes in patients enrolled in the Malmö Diet and Cancer Study (MDCS).

**Patients and methods:**

Circulating lipid levels were measured in blood sampled upon enrollment in the female MDCS cohort (*N* = 17,035). We identified all MDCS participants with incident invasive breast cancer diagnosed between 1991 and 2014. Follow-up time began at breast cancer diagnosis and continued until the first event of breast cancer recurrence, death, emigration, or 5 years of follow-up. We estimated the incidence rates of recurrence at 5 years and fit Cox regression models to compute crude and adjusted hazard ratios (HRs) with 95% confidence intervals (95% CI) of breast cancer recurrence as well as all-cause mortality according to cohort-specific tertiles of apolipoprotein A-1 (Apo A-1) and apolipoprotein B (Apo B).

**Results:**

We enrolled 850 eligible patients. During the 5 years of follow-up, 90 invasive breast cancer recurrences were diagnosed over 3807 person-years. In multivariable analyses, high baseline levels of Apo B were associated with an increased rate of recurrence (tertile 3 vs. 1, HR = 2.30 [95% CI 1.13–4.68]). However, high baseline levels of Apo B were not associated with all-cause mortality (tertile 3 vs. 1, HR = 1.23 [95% CI 0.68–2.25]). We observed no associations between levels of Apo A-1 and recurrence (tertile 3 vs. 1, HR = 1.34 [95% CI 0.70–2.58]) or all-cause mortality (tertile 3 vs. 1, HR = 1.12 [95% CI 0.61–2.05]).

**Conclusion:**

High pre-diagnostic levels of Apo B were associated with an increased risk of recurrence among breast cancer patients. Circulating Apo A-1 was not associated with breast cancer outcomes.

## Introduction

About 2.1 million women are diagnosed with breast cancer worldwide every year [1]. The survival rates for breast cancer have improved over the last 30 years [2]. However, around 30% of the women who are declared breast cancer free after primary therapy will eventually present with a recurrence [3]. To improve breast cancer outcomes, there is a need for identifying modifiable causes of breast cancer recurrence (BCR) and breast cancer-related mortality (BCRM) [4].

A meta-analysis of 82 studies concluded that a higher degree of adiposity—a potential modifiable risk factor—is associated with increased risk of BCR and BCRM [5]. Levels of circulating lipids are altered in obese subjects relative to lean controls, and the association between obesity and risk of BCR and BCRM may be partially explained by alterations in circulating lipid levels [6]. An earlier study reported an association between dyslipidemia (defined as abnormal serum levels of total cholesterol and triglycerides) and increased risk of BCRM [7, 8]. Breast cancer cell lines demonstrate alterations in their lipid metabolism compared with normal breast epithelial cells [9, 10]. This may follow from a greater need for structural lipids in the synthesis of membranes, lipid signaling, and activation of inflammation-related pathways to support tumor survival [11]. The changes in lipid metabolism in cancer are all related to cell growth, proliferation, differentiation, and motility [11].

Cholesterol is an essential lipid for the synthesis of cell membranes [12]. High plasma cholesterol levels have a well-established role in cardiovascular diseases and are associated with an increased risk of and poor prognosis in breast cancer [13]. Cholesterol is transported in the blood via low-density lipoprotein (LDL) and high-density lipoprotein (HDL) [14]; levels of both LDL and HDL have been associated with worse prognosis in breast cancer patients [15, 16]. However, the results are conflicting [15–18] and need to be further explored. Due to differences in their surface, these lipoproteins contain different classes of apolipoproteins. Apolipoprotein A-1 (Apo A-1) is the most abundant apolipoprotein in HDL [19]. Apo A-1 plays a role in the release of cholesterol from cells and has anti-inflammatory, anti-oxidant, and anti-apoptotic properties [20]. Studies have found that the cholesterol metabolite 27-hydroxycholesterol (27-HC) is increased when Apo A-1 is overexpressed in mouse models of metastatic breast cancer [21]. Of note, 27-HC is associated with an increase in breast tumor growth and metastasis in estrogen-receptor-positive breast cancer [22]. The role of Apo A-1 in breast cancer has been debated, and the results are conflicting [23–26]. Apolipoprotein B (Apo B) is the major component of LDL [27] and has been well-studied with regard to cardiovascular diseases [28]. In cancer, experimental studies have linked low tumor expression of Apo B to an increase in metastatic and oncogenic regulators and inhibition of tumor suppressors [29]. Furthermore, high levels of circulating Apo B are associated with diabetes [30], atherogenic disorders [31], and metabolic syndrome [32]; these are all diseases that can affect cancer progression [33]. The association between circulating Apo B levels and breast cancer initiation has been investigated with conflicting results [25, 34], but no studies have investigated the link between circulating levels of Apo B and cancer progression.

This study investigated the prognostic role of pre-diagnostic circulating Apo A-1 and Apo B in breast cancer and provides further insight into the interplay between circulating lipids and breast cancer progression. We hypothesized that, compared with low levels of circulating Apo A-1 and Apo B, respectively, high levels of circulating Apo A-1 would be associated with better prognosis of breast cancer, and that high levels of circulating Apo B would be associated with worse prognosis.

## Patients and methods

We conducted a prospective cohort study nested in the Malmö Diet and Cancer Study (MDCS) cohort [35].

### Data sources

The MDCS is a prospective cohort study that enrolled 17,035 women in Malmö, Sweden from 1991 to 1996 [35]. The primary objective of the MDCS was to investigate associations between dietary patterns and cancer risk. The women studied were born between 1923 and 1950 and the MDCS successfully enrolled 42.6% of the eligible geographic population [36]. Women were not included if they had limited Swedish language skills or disabilities prohibiting completion of study questionnaires. The participants answered extensive questionnaires at baseline and blood samples were collected. Trained research nurses obtained anthropometric measurements including height, weight, and waist/hip circumferences. The MDCS is updated annually with information on incident cancer cases and vital status through record linkage to the Swedish Cancer Registry, the Southern Swedish Regional Tumor Registry, and the Swedish Cause of Death Registry [36]. In the National Population Register, a unique civil registration number is assigned to all Swedish residents, which allows the data sources to be linked with 100% accuracy.

Drug data were retrieved from the Swedish Prescription Registry [37], which collected information on filled prescriptions including anatomical therapeutic chemical codes (ATC) and the date of dispensing at hospitals and pharmacies in Sweden since July 2005. Follow-up data were retrieved from medical records, the Swedish Cause of Death Registry [38], and the Swedish Population Register [39].

### Study population

**Fig. 1:**
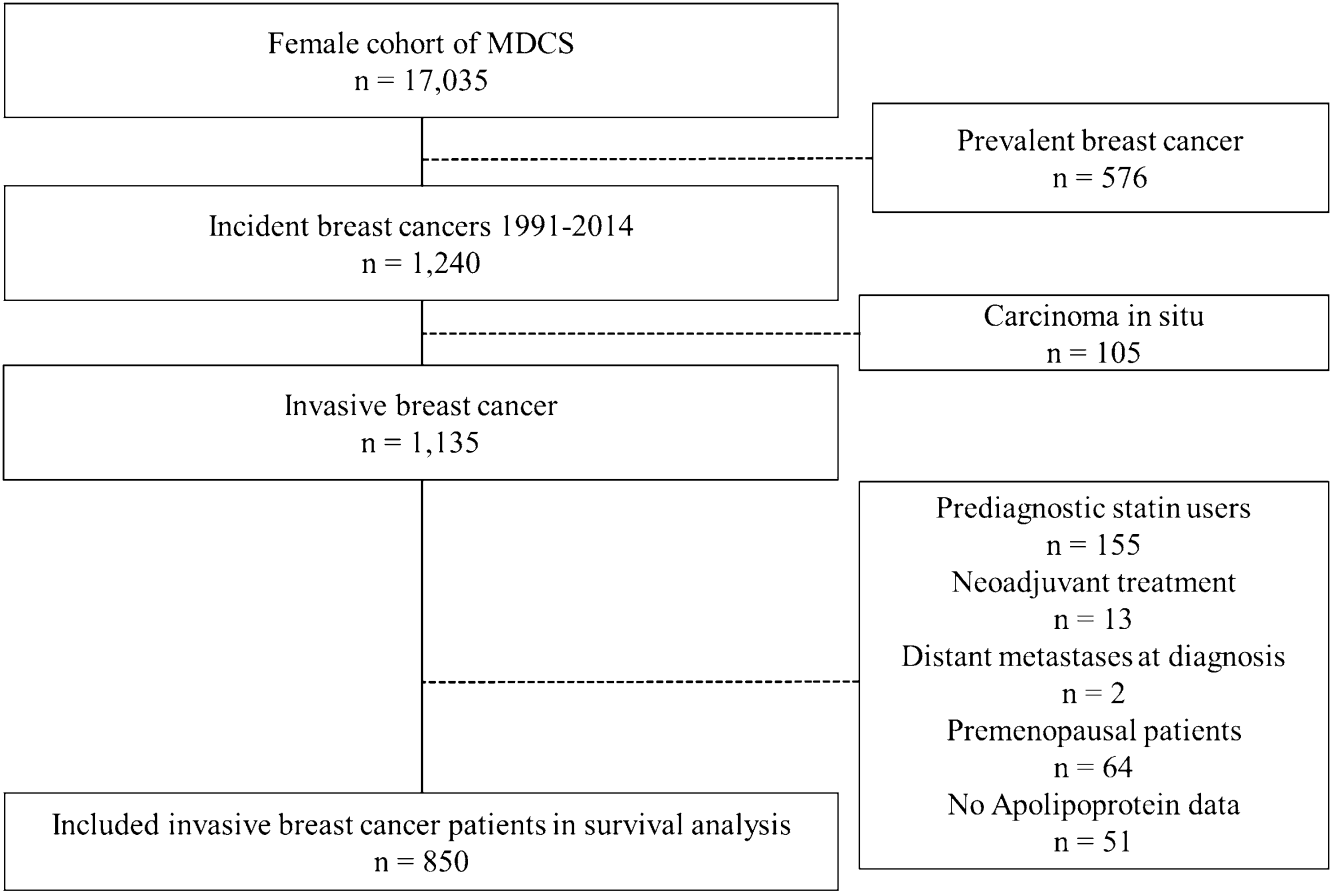
Flowchart of the study cohort

We identified 1240 female MDCS participants with an incident diagnosis of breast cancer between 1991 and 2014 (Fig. 1). We excluded patients with a breast cancer diagnosis before enrollment in MDCS and patients diagnosed with incident breast carcinoma in situ. Patients were also excluded if they had received neoadjuvant treatment or if metastases were present at breast cancer diagnosis. Likewise, premenopausal patients and patients prescribed cholesterol-lowering statins before their breast cancer diagnosis were excluded because the menopausal transition and statin use both alter apolipoprotein levels. Finally, patients had to have circulating blood lipid levels measured to be included in the survival analyses. After exclusion, 850 patients were included in the survival analysis (Fig. 1).

### Apolipoprotein profiles

Blood samples were retrieved from non-fasting patients at enrollment in the MDCS. Serum and plasma were separated within one hour and stored at − 80 °C; serum concentrations of Apo A-1 and Apo B were measured by Quest Diagnostics (*San Juan Capistrano, CA*) using an immunonephelometric assay run on the Siemens BNII (*Siemens, Newark, DE*). The technicians were blinded to cancer status. The inter-assay variability for Apo A-1 and Apo B was below 4%. Further details about the treatment of blood samples have been described previously [35, 40].

### Definitions of analytic variables

We grouped Apo A-1 into tertiles according to the distribution of blood levels in the study-specific cohort of MDCS: tertile 1 (< 154 mg/dl), tertile 2 (154–177 mg/dl), and tertile 3 (> 177 mg/dl). These groups were modeled as categorical variables with the lowest tertile as the reference group. The same approach was used to define tertiles of Apo B: tertile 1 (< 90 mg/dl), tertile 2 (90–110 mg/dl), and tertile 3 (> 110 mg/dl). Likewise, Apo B was included in the models as a categorical variable with the lowest tertile as the reference group. Patients in tertile 1 had low levels, patients in tertile 2 had medium levels, and patients in tertile 3 had high levels of either Apo A-1 or Apo B.

Age was described in decade categories at enrollment in MDCS. In the multivariable models, age was included as a continuous variable defined at the time of breast cancer diagnosis. We used the World Health Organization’s body mass index (BMI) definitions [41]: normal-weight (BMI < 25 kg/m^2^), overweight (BMI ≥ 25 and < 30 kg/m^2^), obese (BMI ≥ 30 kg/m^2^). BMI was included in the regression models as a continuous variable and finer adjustment with factor variables did not affect the impact of adjustment for BMI on the main exposure term. Patients were further described by three categories of waist circumference in centimeters (< 81, 81–85, and > 85) and waist/hip ratio (≤ 0.80, 0.81–0.84, and ≥ 0.85).

We defined three categories of tumor size in millimeters (mm): less than 10 mm, 10–20 mm, and more than 20 mm [42]; tumor size was modeled as a categorical variable. The histological grade of breast cancers was assessed and described according to the Nottingham histological score [43]. Histological grade was categorized as grade I (well differentiated [3–5 points]), grade II (moderately differentiated [6–7 points]), and grade III (poorly differentiated [8–9 points]); this was modeled as a categorical variable. Post-diagnostic statin use was included in the model as a dichotomous time-varying variable, and one filled prescription for statins was assumed to expose the individual from the prescription date through the remaining follow-up time. Estrogen-receptor status (positive/negative), lymph node status (positive [present metastatic lymph nodes]/negative [no metastatic lymph nodes]), surgery (lumpectomy/mastectomy) as well as intended adjuvant treatment with radiotherapy (yes/no), chemotherapy (yes/no), and endocrine therapy (yes/no) were treated as dichotomous variables.

### Outcome

Our primary endpoint was breast cancer recurrence, defined as time from breast cancer diagnosis until the earliest occurrence of invasive loco-regional recurrence, distant metastasis, invasive contralateral breast cancer, or second primary breast cancer. Distant recurrence was defined as time from breast cancer diagnosis until occurrence of metastasis, invasive contralateral breast cancer, or second primary breast cancer. Loco-regional recurrence was defined as time from breast cancer diagnosis until occurrence of recurrence without signs of metastatic disease. Trained personnel retrieved data on recurrence from medical charts of all patients diagnosed with breast cancer. Our secondary endpoint was all-cause mortality. Information regarding cause of death was retrieved using the Swedish Cause of Death Registry and defined as time from breast cancer diagnosis until death.

### Follow-up and statistical analysis

Follow-up for breast cancer recurrence and all-cause mortality began at the time of breast cancer diagnosis and continued until the first event of breast cancer recurrence, death from any cause, emigration, or 5 years of follow-up. Patients with these events were censored at the time of event.

We estimated incidence rates of BCR at 5 years and used Cox regression models to compute crude and adjusted hazard ratios (HRs) with 95% confidence intervals (95% CI) for both breast cancer recurrence (BCR) and all-cause mortality. Only patients with complete data on all regressed variables were included in the analyses. The adjusted model included the following covariables: age at diagnosis (continuous), body mass index (continuous), node status (dichotomous), estrogen-receptor status (dichotomous), histological grade (categorical), tumor size (categorical), adjuvant radiotherapy (dichotomous), adjuvant chemotherapy (dichotomous), adjuvant endocrine therapy (dichotomous), type of surgery (dichotomous), and post-diagnostic use of statins (dichotomous, time-varying). In the crude analyses, 850 patients were included. Complete case data were available for 650 patients, which were included in the adjusted models. We conducted a series of sensitivity analyses. First, we examined possible differences in types of BCR using Cox regression models to compute HRs and 95% CI of loco-regional recurrence and distant recurrence. Second, as a large proportion of breast cancers recur late [44], we performed analyses where follow-up beyond 5 years was allowed. Finally, to examine possible differences in the association between apolipoproteins and breast cancer prognosis according to patterns of breast cancer detection, we conducted analyses stratified for the detection of breast cancer through screening mammography or clinical detection.

## Results

In the cohort of 850 patients with incident postmenopausal breast cancer, the median age at diagnosis was 56.3 years (interquartile range was 50.3–62.4). Most breast cancers in this study were detected through mammography screening (screening-detected, *n* = 436 vs clinically detected, *n* = 406). The median recurrence follow-up was 5 years and the total person-years of recurrence follow-up was 3807, during which 90 recurrences occurred: 11 loco-regional and 79 distant.

Patients with high levels of Apo A-1 appeared more likely to have axillary lymph node involvement at diagnosis versus patients with low Apo A-1 levels. Further, patients with high levels of Apo A-1 were more frequently normal-weight, had a smaller waist circumference, and a lower waist/hip ratio. Most patients underwent lumpectomy, however, patients with high levels of Apo A-1 were more frequently appointed mastectomy. Patients with high levels of Apo A-1 were also less frequently prescribed statins (Table 1).

**Table 1:** Patient characteristics according to Apolipoprotein A-1 tertiles

	Total	1 (< 154 mg/dl)	2 (154–177 mg/dl)	3 (> 177 mg/dl)
	*N* = 850	*N* = 292	*N* = 286	*N* = 272
Age (baseline)				
< 50	193 (22.7%)	78 (26.7%)	59 (20.6%)	56 (20.6%)
50–59	359 (42.2%)	118 (40.4%)	122 (42.7%)	119 (43.8%)
60–69	243 (28.6%)	74 (25.3%)	81 (28.3%)	88 (32.4%)
≥ 70	55 (6.5%)	22 (7.5%)	24 (8.4%)	9 (3.3%)
Histological grade				
Grade I	222 (28.5%)	82 (30.6%)	67 (25.3%)	73 (29.6%)
Grade II	363 (46.5%)	116 (43.3%)	132 (49.8%)	115 (46.6%)
Grade III	195 (25.0%)	70 (26.1%)	66 (24.9%)	59 (23.9%)
Tumor size (mm)				
< 10	151 (18.6%)	53 (19.1%)	47 (17.2%)	51 (19.5%)
10–20	435 (53.6%)	140 (50.5%)	151 (55.3%)	144 (55.0%)
> 20	226 (27.8%)	84 (30.3%)	75 (27.5%)	67 (25.6%)
Node status				
Negative	526 (69.3%)	175 (68.9%)	190 (72.8%)	161 (66.0%)
Positive	233 (30.7%)	79 (31.1%)	71 (27.2%)	83 (34.0%)
Estrogen-receptor status				
Negative	93 (12.3%)	24 (9.3%)	37 (14.2%)	32 (13.4%)
Positive	663 (87.7%)	233 (90.7%)	224 (85.8%)	206 (86.6%)
Body mass index				
Normal-weight	436 (51.3%)	122 (41.8%)	144 (50.3%)	170 (62.5%)
Overweight	298 (35.1%)	119 (40.8%)	96 (33.6%)	83 (30.5%)
Obese	116 (13.6%)	51 (17.5%)	46 (16.1%)	19 (7.0%)
Waist/hip ratio				
≤ 0.80	531 (62.5%)	159 (54.6%)	175 (61.2%)	197 (72.4%)
0.81–0.84	205 (24.1%)	83 (28.5%)	67 (23.4%)	55 (20.2%)
≥ 0.85	113 (13.3%)	49 (16.8%)	44 (15.4%)	20 (7.4%)
Waist circumference (cm)				
< 81	563 (66.3%)	166 (57.0%)	187 (65.4%)	210 (77.2%)
81–85	109 (12.8%)	52 (17.9%)	26 (9.1%)	31 (11.4%)
> 85	177 (20.8%)	73 (25.1%)	73 (25.5%)	31 (11.4%)
Surgical procedure				
Lumpectomy	465 (59.3%)	163 (59.5%)	162 (63.3%)	140 (55.1%)
Mastectomy	319 (40.7%)	111 (40.5%)	94 (36.7%)	114 (44.9%)
Endocrine therapy, planned adjuvant				
No	363 (44.3%)	122 (43.9%)	123 (44.7%)	118 (44.4%)
Yes	456 (55.7%)	156 (56.1%)	152 (55.3%)	148 (55.6%)
Chemotherapy, planned adjuvant				
No	659 (85.5%)	222 (85.1%)	231 (88.8%)	206 (82.4%)
Yes	112 (14.5%)	39 (14.9%)	29 (11.2%)	44 (17.6%)
Radiotherapy, planned adjuvant				
No	323 (41.7%)	109 (41.8%)	105 (40.1%)	109 (43.3%)
Yes	452 (58.3%)	152 (58.2%)	157 (59.9%)	143 (56.7%)
Postdiagnostic statin use				
No	751 (88.4%)	249 (85.3%)	254 (88.8%)	248 (91.2%)
Yes	99 (11.6%)	43 (14.7%)	32 (11.2%)	24 (8.8%)

Patients with high levels of Apo B had higher histological grade and larger tumors. In contrast to patients with high levels of Apo A-1, patients with high levels of Apo B were older and more often overweight or obese. Patients with high Apo B levels were also more often appointed lumpectomy and more frequently prescribed statins than patients with low levels of Apo B (Table 2).

**Table 2:** Patient characteristics according to Apolipoprotein B tertiles

	Total	1 (< 90 mg/dl)	2 (90–110 mg/dl)	3 (> 110 mg/dl)
	*N* = 850	*N* = 286	*N* = 284	*N* = 280
Age (baseline)				
< 50	193 (22.7%)	104 (36.4%)	62 (21.8%)	27 (9.6%)
50–59	359 (42.2%)	121 (42.3%)	126 (44.4%)	112 (40.0%)
60–69	243 (28.6%)	56 (19.6%)	71 (25.0%)	116 (41.4%)
≥ 70	55 (6.5%)	5 (1.7%)	25 (8.8%)	25 (8.9%)
Histological grade				
Grade I	222 (28.5%)	88 (34.1%)	69 (26.2%)	65 (25.1%)
Grade II	363 (46.5%)	112 (43.4%)	132 (50.2%)	119 (45.9%)
Grade III	195 (25.0%)	58 (22.5%)	62 (23.6%)	75 (29.0%)
Tumor size (mm)				
< 10	151 (18.6%)	52 (19.0%)	64 (23.4%)	35 (13.3%)
10–20	435 (53.6%)	158 (57.7%)	138 (50.4%)	139 (52.7%)
> 20	226 (27.8%)	64 (23.4%)	72 (26.3%)	90 (34.1%)
Node status				
Negative	526 (69.3%)	189 (73.3%)	179 (69.1%)	158 (65.3%)
Positive	233 (30.7%)	69 (26.7%)	80 (30.9%)	84 (34.7%)
Estrogen-receptor status				
Negative	93 (12.3%)	26 (10.4%)	30 (11.7%)	37 (14.9%)
Positive	663 (87.7%)	225 (89.6%)	226 (88.3%)	212 (85.1%)
Body mass index				
Normal-weight	436 (51.3%)	185 (64.7%)	153 (53.9%)	98 (35.0%)
Overweight	298 (35.1%)	78 (27.3%)	104 (36.6%)	116 (41.4%)
Obese	116 (13.6%)	23 (8.0%)	27 (9.5%)	66 (23.6%)
Waist/hip ratio				
≤ 0.80	531 (62.5%)	212 (74.4%)	185 (65.1%)	134 (47.9%)
0.81–0.84	205 (24.1%)	50 (17.5%)	65 (22.9%)	90 (32.1%)
≥ 0.85	113 (13.3%)	23 (8.1%)	34 (12.0%)	56 (20.0%)
Waist circumference (cm)				
< 81	563 (66.3%)	229 (80.4%)	197 (69.4%)	137 (48.9%)
81–85	109 (12.8%)	27 (9.5%)	41 (14.4%)	41 (14.6%)
> 85	177 (20.8%)	29 (10.2%)	46 (16.2%)	102 (36.4%)
Surgical procedure				
Lumpectomy	465 (59.3%)	163 (61.3%)	164 (61.7%)	138 (54.8%)
Mastectomy	319 (40.7%)	103 (38.7%)	102 (38.3%)	114 (45.2%)
Endocrine therapy, planned adjuvant				
No	363 (44.3%)	119 (43.0%)	131 (47.8%)	113 (42.2%)
Yes	456 (55.7%)	158 (57.0%)	143 (52.2%)	155 (57.8%)
Chemotherapy, planned adjuvant				
No	659 (85.5%)	218 (84.8%)	222 (85.4%)	219 (86.2%)
Yes	112 (14.5%)	39 (15.2%)	38 (14.6%)	35 (13.8%)
Radiotherapy, planned adjuvant				
No	323 (41.7%)	106 (41.2%)	99 (37.9%)	118 (45.9%)
Yes	452 (58.3%)	151 (58.8%)	162 (62.1%)	139 (54.1%)
Postdiagnostic statin use				
No	751 (88.4%)	271 (94.8%)	247 (87.0%)	233 (83.2%)
Yes	99 (11.6%)	15 (5.2%)	37 (13.0%)	47 (16.8%)

**Fig. 2:**
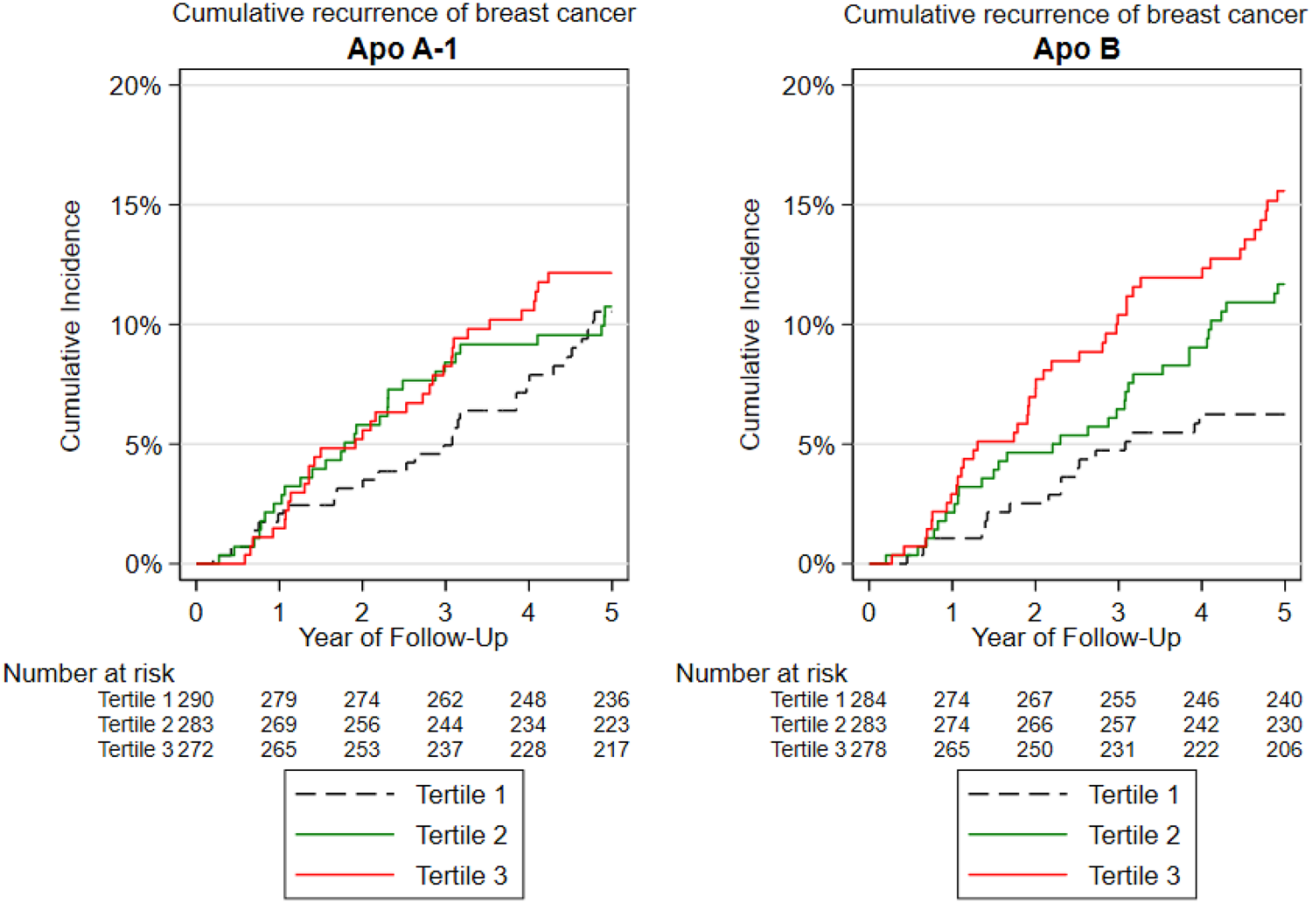
Cumulative incidence of recurrence in breast cancer according to apolipoprotein tertiles in patients enrolled in MDCS

Table 3 presents the estimated recurrence and mortality hazard ratios in relation to Apo A-1 tertiles. There were 29 recurrences in tertile 1 (T1) during 1324 person-years of follow-up, 29 recurrences in tertile 2 (T2) in 1257 person-years, and 32 recurrences occurred in 1224 person-years of follow-up in tertile 3 (T3). No strong associations were seen between levels of Apo A-1 and BCR (T3, _adj_HR: 1.34 [95% CI 0.70–2.58]), distant recurrence (T3, _adj_HR: 1.29 [95% CI 0.66–2.50]), loco-regional recurrence (T3, _adj_HR: 1.19 [95% CI 0.30–4.75]), or all-cause mortality (T3, _adj_HR: 1.12 [95% CI 0.61–2.05]) (Table 3, Fig. 2). Neither were any association between Apo A-1 and BCR observed in analyses stratified for detection mode of breast cancer through mammography screening (T3, _adj_HR: 1.06 [95% CI 0.36–3.15]) or clinical detection (T3, _adj_HR: 1.40 [95% CI 0.59–3.34]) (Table 5).

**Table 3:** Apolipoprotein A-1 tertiles in relation to recurrence and survival

Endpoint	Tertiles	Events	Person-years	Unadjusted HR (95% CI)	Adjusted HR (95% CI)^a^
Any recurrence					
	T1	29	1324.83	(Ref)	
	T2	29	1257.82	1.05 (0.62–1.78)	1.20 (0.62–2.32)
	T3	32	1224.85	1.19 (0.72–1.99)	1.34 (0.70–2.58)
Distant recurrence					
	T1	25	356.49	(Ref)	
	T2	27	370.52	1.14 (0.65–1.97)	1.20 (0.62–2.32)
	T3	28	428.93	1.25 (0.73–2.16)	1.26 (0.65–2.45)
All-cause mortality					
	T1	35	1135.72	(Ref)	
	T2	45	860.90	1.34 (0.86–2.08)	1.11 (0.63–1.98)
	T3	39	923.52	1.18 (0.75–1.87)	1.12 (0.61–2.05)

^a^Adjusted for age, body mass index, estrogen-receptor status, histological grade, nodal status, tumor size, adjuvant endocrine treatment, chemotherapy, radiotherapy, surgery and statins

Table 4 displays estimated recurrence and mortality hazard ratios in relation to Apo B tertiles. There were 17 recurrences among patients in T1 during 1304 person-years of follow-up, 32 recurrences in T2 in 1293 person-years, and 41 recurrences in 1209 person-years of follow-up in T3. In univariable analyses, high levels of Apo B were associated with BCR (T3, _crude_HR: 2.69 [95% CI 1.51–4.81]) and distant recurrence (T3, _crude_HR: 2.61 [95% CI 1.40–4.87]). High levels of Apo B were also positively associated with BCR (T3, _adj_HR: 2.30 [95% CI 1.13–4.68]) and distant recurrence (T3, _adj_HR: 2.19 [95% CI 1.02–4.70]) in multivariable analyses. However, high levels of Apo B were not associated with an increased risk of loco-regional recurrence (T3, _adj_HR: 0.73 [95% CI 0.16–3.27]) or all-cause mortality (T3, _adj_HR: 1.23 [95% CI 0.68–2.25]) (Table 4, Fig. 2). In analyses allowing a longer follow-up than 5 years, the positive association between high levels of Apo B and BCR was consistent (T3, _adj_HR: 1.70 [95% CI 1.0–2.88]). When analyses were stratified for primary breast cancer mode of detection, the association between Apo B and BCR was attenuated for patients detected through mammography screening (T3, _adj_HR: 1.01 [95% CI 0.31–3.28]), while the association was persistent for patients with a clinically detected breast cancer (T3, _adj_HR: 3.87 [95% CI 1.39–10.75]) (Table 5).

**Table 4:** Apolipoprotein B tertiles in relation to recurrence and survival

Endpoint	Tertiles	Events	Person-years	Unadjusted HR (95% CI)	Adjusted HR (95% CI)^a^
Any recurrence					
	T1	17	1304.19	(Ref)	
	T2	32	1293.50	1.96 (1.07–3.58)	1.70 (0.82–3.53)
	T3	41	1209.82	2.69 (1.51–4.81)	2.30 (1.13–4.68)
Distant recurrence					
	T1	15	348.64	(Ref)	
	T2	30	448.68	2.15 (1.14–4.06)	1.61 (0.74–3.52)
	T3	35	358.62	2.61 (1.40–4.87)	2.19 (1.02–4.70)
All-cause mortality					
	T1	35	670.84	(Ref)	
	T2	34	1033.77	0.97 (0.61–1.56)	0.81 (0.42–1.56)
	T3	50	1215.53	1.52 (0.98–2.34)	1.23 (0.68–2.25)

^a^Adjusted for age, body mass index, estrogen-receptor status, histological grade, nodal status, tumor size, adjuvant endocrine treatment, chemotherapy, radiotherapy, surgery and statins

**Table 5:** Primary detection and apolipoproteins in relation to recurrence

Primary breast cancer	Tertiles	Apo A-1			Apo B		
		Events	Person-years	Unadjusted HR (95% CI)	Events	Person-years	Unadjusted HR (95% CI)
Screening detected							
	T1	11	677.99	(Ref)	9	673.98	(Ref)
	T2	10	584.03	1.06 (0.45–2.49)	10	653.91	1.14 (0.47–2.82)
	T3	12	672.58	1.10 (0.49–2.50)	14	606.71	1.73 (0.75–4.01)
Clinically detected							
	T1	13	552.16	(Ref)	6	587.48	(Ref)
	T2	16	599.01	1.14 (0.55–2.36)	20	546.71	3.57 (1.43–8.90)
	T3	19	480.81	1.67 (0.82–3.38)	22	497.79	4.31 (1.75–10.64)

Primary breast cancer	Tertiles	Apo A-1			Apo B		
		Events	Person-years	Adjusted HR (95% CI)*	Events	Person-years	Adjusted HR (95% CI)^a^
Screening detected							
	T1	11	677.99		9	673.98	
	T2	10	584.03	0.51 (0.16–1.68)	10	653.91	0.54 (0.15–1.90)
	T3	12	672.58	1.06 (0.36–3.15)	14	606.71	1.01 (0.31–3.28)
Clinically detected							
	T1	13	552.16		6	587.48	
	T2	16	599.01	1.98 (0.84–4.71)	20	546.71	3.40 (1.19–9.72)
	T3	19	480.81	1.40 (0.59–3.34)	22	497.79	3.87 (1.39–10.75)

^a^Adjusted for age, body mass index, estrogen-receptor status, histological grade, nodal status, tumor size, adjuvant endocrine treatment, chemotherapy, radiotherapy, surgery and statins

## Discussion

This observational study showed a positive association between high levels of pre-diagnostic Apo B and breast cancer recurrence. This association remained positive for distant recurrences but not for loco-regional recurrences. In the 5-year estimates, patients with high levels of Apo B had a two-fold increased risk of recurrence of their breast cancer versus patients with low levels of Apo B. In contrast, there was no association between Apo A-1 levels and breast cancer outcomes. To the best of our knowledge, this is the first study to evaluate if Apo A-1 and Apo B are associated with recurrence in breast cancer patients.

Our study has some limitations. First, the measurements of circulating apolipoprotein levels were obtained from baseline serum specimens and were drawn well before breast cancer diagnosis. This might induce a misclassification of apolipoprotein levels at etiologically appropriate time periods because apolipoprotein levels can be modulated, e.g., by lifestyle changes, statins, or entering menopause. To minimize the risk of misclassification and time-dependent confounding, we excluded all premenopausal patients and patients who were prescribed statins before their breast cancer diagnosis. However, we cannot exclude the possibility that lipid levels may have changed between blood draw and breast cancer diagnosis; our findings may therefore still be prone to misclassification.

Second, many patients received adjuvant endocrine therapy, and endocrine therapy might modulate circulating apolipoprotein levels differently, e.g., tamoxifen increases Apo A-1 [45] and aromatase inhibitors increase Apo B [46]. Thus, it could have been of value to account for changes in blood levels of apolipoproteins during endocrine therapy. However, this was unfortunately not possible because post-diagnostic blood samples (for Apo A-1 and Apo B analysis) and data on changes in endocrine therapy during follow-up were not available. In a sensitivity analysis stratified by planned endocrine therapy, the positive association between high levels of Apo B and recurrence was only observed for patients receiving endocrine therapy (T3, HR: 3.30 [95% CI 1.22–8.92]).

Third, apolipoprotein levels follow systemic cholesterol levels, and high levels of cholesterol derivatives are associated with poor breast cancer prognosis [22]. The association between Apo B and increased risk of BCR might therefore be driven by other harmful prognostic factors correlated with Apo B such as high blood levels of LDL that have previously been reported to be associated with worse disease-free survival in breast cancer [15]. Blood levels of total cholesterol, HDL and LDL were only collected for a small sub-population in MDCS. However, the size of the sub-population limits statistical analyses and assessment of their importance concerning prognosis in this study.

Finally, it should be acknowledged that there might exist positive confounders, which we were not able to adjust for in our statistical models. Hence, our results might be affected by residual confounding as the association between Apo B and breast cancer recurrence was attenuated in multivariable models.

Apo B’s relation to 27-HC and LDL might explain the potential mechanism linking Apo B levels to a worse outcome in breast cancer—27-HC is primarily transported by LDL and is associated with an increase in breast tumor growth and metastasis in estrogen-receptor-positive breast cancer [22]. Apo B is the main protein constituent of LDL and Apo B thus mirrors LDL levels in serum. The negative prognostic value of high Apo B levels might therefore be explained by the function of Apo B levels as a surrogate marker of 27-HC levels via LDL. Yet, investigating possible differences in Apo B’s association with BCR according to estrogen-receptor status did not explain the association further. Thus, the positive association between Apo B and BCR was not considered limited to a certain subtype of breast cancer in this study.

The difference in association between Apo B levels and outcome observed among patients with primary breast cancer detected through mammography screening and clinical detection may partially be explained by the relationship between Apo B and breast density. Breast density is linked to a higher risk of breast cancer [47], at the same time high breast density makes breast cancers harder to detect through mammography [48]. Previous research has shown an association between low levels of Apo B and high breast density [49]. This would suggest that patients with high levels of Apo B are to be diagnosed with breast cancer earlier than those with low levels as these breast cancers would be harder to identify through mammography screening. Yet, in this study, patients with high levels of Apo B are diagnosed with higher histological grade and larger tumors, which refutes the aforementioned hypothesis.

Measuring Apo B in blood is a part of routine cardiovascular care. It can detect if a patient is at risk of developing cardiovascular disease. We show here an association between Apo B and breast cancer outcome, and the second most common cause of death in breast cancer patients is cardiovascular disease [50]. Thus, it is needed to further investigate the role of apolipoproteins in breast cancer progression in order to answer the question if it is clinically relevant to assay for Apo B levels to predict risk of both BCR and cardiovascular disease in breast cancer patients. Future studies should longitudinally measure blood apolipoprotein levels after breast cancer diagnosis to detect possible changes in lipid metabolism by breast cancer while accounting for tumor, treatment, and patient characteristics.

## Conclusion

High pre-diagnostic levels of Apo B were associated with an increased risk of recurrence among breast cancer patients. Circulating Apo A-1 was not associated with breast cancer outcome. The results warrant further study into the importance of lipid regulation in breast cancer patients.

## Data Availability

The data that support the findings of this study are available to appropriate academic parties upon reasonable request to the *Malmö Preventative Project/ Malmö Diet and Cancer Study/ Malmö Offspring Study* steering committee (please see, https://www.malmo-kohorter.lu.se/malmo-cohorts).

## Abbreviations

Apo A-1: Apolipoprotein A-1
Apo B: Apolipoprotein B
BCR: Breast cancer recurrence
BCRM: Breast cancer-related mortality
LDL: Low-density lipoprotein
HDL: High-density lipoprotein
27-HC: 27-Hydroxycholesterol
MDCS: Malmö diet and cancer study
ATC: Anatomical therapeutic chemical codes
BMI: Body mass index
T1: Tertile 1
T2: Tertile 2
T3: Tertile 3
HR: Hazard ratio
CI: Confidence interval

## Code availability

The code developed during this study is available upon reasonable request. Analyses were performed using Stata®, version 16 (StataCorp, College Station, Texas, USA).

## References

[1] Bray F, Ferlay J, Soerjomataram I, Siegel RL, Torre LA, Jemal A (2018). Global cancer statistics 2018: GLOBOCAN estimates of incidence and mortality worldwide for 36 cancers in 185 countries. CA Cancer J Clin.

[2] DeSantis CE, Ma J, Sauer AG, Newman LA, Jemal A (2017). Breast cancer statistics, 2017, racial disparity in mortality by state. CA Cancer J Clin.

[3] Saphner T, Tormey DC, Gray R (1996). Annual hazard rates of recurrence for breast cancer after primary therapy. J Clin Oncol Off J Am Soc Clin Oncol.

[4] Lafourcade A, His M, Baglietto L, Boutron-Ruault M-C, Dossus L, Rondeau V (2018). Factors associated with breast cancer recurrences or mortality and dynamic prediction of death using history of cancer recurrences: the French E3N cohort. BMC Cancer.

[5] Chan DSM, Vieira AR, Aune D, Bandera EV, Greenwood DC, McTiernan A (2014). Body mass index and survival in women with breast cancer-systematic literature review and meta-analysis of 82 follow-up studies. Ann Oncol Off J Eur Soc Med Oncol.

[6] Ren L, Yi J, Li W, Zheng X, Liu J, Wang J (2019). Apolipoproteins and cancer. Cancer Med.

[7] Emaus A, Veierød MB, Tretli S, Finstad SE, Selmer R, Furberg A-S (2010). Metabolic profile, physical activity, and mortality in breast cancer patients. Breast Cancer Res Treat.

[8] Buono G, Crispo A, Giuliano M, De Angelis C, Schettini F, Forestieri V (2020). Metabolic syndrome and early stage breast cancer outcome: results from a prospective observational study. Breast Cancer Res Treat.

[9] Oncoscience | The metastasis inducer CCN1 (CYR61) activates the fatty acid synthase (FASN)-driven lipogenic phenotype in breast cancer cells [Internet]. https://www.oncoscience.us/article/314/text/www.oncoscience.us/article/314/text/. Accessed 3 Aug 2021 10.18632/oncoscience.314 PMC5043073 27713913

[10] Schug ZT, Peck B, Jones DT, Zhang Q, Grosskurth S, Alam IS (2015). Acetyl-CoA synthetase 2 promotes acetate utilization and maintains cancer cell growth under metabolic stress. Cancer Cell.

[11] Hilvo M, Orešiè AM (2012). Regulation of lipid metabolism in breast cancer provides diagnostic and therapeutic opportunities. Clin Lipidol.

[12] Huff T, Boyd B, Jialal I (2020) Physiology, Cholesterol. I: StatPearls [Internet]. Treasure Island (FL): StatPearls Publishing. http://www.ncbi.nlm.nih.gov/books/NBK470561/. Accessed 10 Feb 2021

[13] Nelson ER, Chang C, McDonnell DP (2014). Cholesterol and breast cancer pathophysiology. Trends Endocrinol Metab.

[14] Feingold KR, Grunfeld C (2000) Introduction to lipids and lipoproteins. In: Feingold KR, Anawalt B, Boyce A, Chrousos G, de Herder WW, Dungan K et al. (eds) Endotext [Internet]. MDText.com, Inc., South Dartmouth (MA). http://www.ncbi.nlm.nih.gov/books/NBK305896/. Accessed 1 Feb 2021

[15] Rodrigues dos Santos C, Fonseca I, Dias S, Mendes de Almeida J (2014). Plasma level of LDL-cholesterol at diagnosis is a predictor factor of breast tumor progression. BMC Cancer.

[16] Li X, Tang H, Wang J, Xie X, Liu P, Kong Y (2017). The effect of preoperative serum triglycerides and high-density lipoprotein-cholesterol levels on the prognosis of breast cancer. The Breast.

[17] Lofterød T, Mortensen ES, Nalwoga H, Wilsgaard T, Frydenberg H, Risberg T (2018). Impact of pre-diagnostic triglycerides and HDL-cholesterol on breast cancer recurrence and survival by breast cancer subtypes. BMC Cancer.

[18] Jung SM, Kang D, Guallar E, Yu J, Lee JE, Kim SW (2020). Impact of serum lipid on breast cancer recurrence. J Clin Med.

[19] Lee-Rueckert M, Escola-Gil JC, Kovanen PT (2016). HDL functionality in reverse cholesterol transport–Challenges in translating data emerging from mouse models to human disease. Biochim Biophys Acta.

[20] Georgila K, Vyrla D, Drakos E (2019). Apolipoprotein A-I (ApoA-I), immunity, inflammation and cancer. Cancers.

[21] Cedó L, García-León A, Baila-Rueda L, Santos D, Grijalva V, Martínez-Cignoni MR (2016). ApoA-I mimetic administration, but not increased apoA-I-containing HDL, inhibits tumour growth in a mouse model of inherited breast cancer. Sci Rep.

[22] Kimbung S, Chang C, Bendahl P-O, Dubois L, Thompson JW, McDonnell DP (2017). Impact of 27-hydroxylase (CYP27A1) and 27-hydroxycholesterol in breast cancer. Endocr Relat Cancer.

[23] Chang S-J, Hou M-F, Tsai S-M, Wu S-H, Hou LA, Ma H (2007). The association between lipid profiles and breast cancer among Taiwanese women. Clin Chem Lab Med.

[24] His M, Zelek L, Deschasaux M, Pouchieu C, Kesse-Guyot E, Hercberg S (2014). Prospective associations between serum biomarkers of lipid metabolism and overall, breast and prostate cancer risk. Eur J Epidemiol.

[25] Borgquist S, Butt T, Almgren P, Shiffman D, Stocks T, Orho-Melander M (2016). Apolipoproteins, lipids and risk of cancer. Int J Cancer.

[26] Huang H-L, Stasyk T, Morandell S, Dieplinger H, Falkensammer G, Griesmacher A (2006). Biomarker discovery in breast cancer serum using 2-D differential gel electrophoresis/MALDI-TOF/TOF and data validation by routine clinical assays. Electrophoresis.

[27] Devaraj S, Semaan JR, Jialal I (2020) Biochemistry, apolipoprotein B. I: StatPearls [Internet]. StatPearls Publishing, Treasure Island (FL). http://www.ncbi.nlm.nih.gov/books/NBK538139/. 1 Feb 2021 30844166

[28] Contois JH, McConnell JP, Sethi AA, Csako G, Devaraj S, Hoefner DM (2009). Apolipoprotein B and cardiovascular disease risk: position statement from the AACC Lipoproteins and Vascular Diseases Division Working Group on Best Practices. Clin Chem. Marts.

[29] Lee G, Jeong YS, Kim DW, Kwak MJ, Koh J, Joo EW (2018). Clinical significance of APOB inactivation in hepatocellular carcinoma. Exp Mol Med.

[30] Apolipoprotein B but not LDL cholesterol is associated with coronary artery calcification in type 2 diabetic whites | diabetes [Internet]. https://diabetes.diabetesjournals.org/content/58/8/1887. Accessed 1 Feb 2021 10.2337/db08-1794 PMC2712798 19491209

[31] Sniderman AD, Thanassoulis G, Glavinovic T, Navar AM, Pencina M, Catapano A (2019). Apolipoprotein B particles and cardiovascular disease: a narrative review. JAMA Cardiol.

[32] Lim Y, Yoo S, Lee SA, Chin SO, Heo D, Moon JC (2015). Apolipoprotein B is related to metabolic syndrome independently of low density lipoprotein cholesterol in patients with type 2 diabetes. Endocrinol Metab.

[33] Koene RJ, Prizment AE, Blaes A, Konety SH (2016). Shared risk factors in cardiovascular disease and cancer. Circulation.

[34] Chandler PD, Song Y, Lin J, Zhang S, Sesso HD, Mora S (2016). Lipid biomarkers and long-term risk of cancer in the Women’s Health Study. Am J Clin Nutr.

[35] Berglund G, Elmståhl S, Janzon L, Larsson SA (1993). Design and feasibility. J Intern Med.

[36] Manjer J, Carlsson S, Elmståhl S, Gullberg B, Janzon L, Lindström M (2001). The Malmö Diet and Cancer Study: representativity, cancer incidence and mortality in participants and non-participants. Eur J Cancer Prev Off J Eur Cancer Prev Organ ECP.

[37] Wettermark B, Hammar N, Fored CM, MichaelFored C, Leimanis A, Otterblad Olausson P (2007). The new Swedish Prescribed Drug Register—opportunities for pharmacoepidemiological research and experience from the first six months. Pharmacoepidemiol Drug Saf.

[38] Brooke HL, Talbäck M, Hörnblad J, Johansson LA, Ludvigsson JF, Druid H (2017). The Swedish cause of death register. Eur J Epidemiol.

[39] Ludvigsson JF, Almqvist C, Bonamy A-KE, Ljung R, Michaëlsson K, Neovius M (2016). Registers of the Swedish total population and their use in medical research. Eur J Epidemiol.

[40] Pero RW, Olsson A, Berglund G, Janzon L, Larsson SA, Elmståhl S (1993). The Malmö biological bank. J Intern Med.

[41] Obesity and overweight [Internet]. https://www.who.int/news-room/fact-sheets/detail/obesity-and-overweight. 2021. https://www.who.int/news-room/fact-sheets/detail/obesity-and-overweight. Accessed 16 Nov 2020

[42] Cong L, Liu Q, Zhang R, Cui M, Zhang X, Gao X (2018). Tumor size classification of the 8th edition of TNM staging system is superior to that of the 7th edition in predicting the survival outcome of pancreatic cancer patients after radical resection and adjuvant chemotherapy. Sci Rep.

[43] Bloom HJG, Richardson WW (1957). Histological grading and prognosis in breast cancer. Br J Cancer.

[44] Pan H, Gray R, Braybrooke J, Davies C, Taylor C, McGale P (2017). 20-Year risks of breast-cancer recurrence after stopping endocrine therapy at 5 years. N Engl J Med.

[45] Morales M, Santana N, Soria A, Mosquera A, Ordovás J, Nóvoa J (1996). Effects of tamoxifen on serum lipid and apolipoprotein levels in postmenopausal patients with breast cancer. Breast Cancer Res Treat.

[46] Elisaf MS, Bairaktari ET, Nicolaides C, Kakaidi B, Tzallas CS, Katsaraki A (2001). Effect of letrozole on the lipid profile in postmenopausal women with breast cancer. Eur J Cancer.

[47] McCormack VA, Silva IS (2006). Breast density and parenchymal patterns as markers of breast cancer risk: a meta-analysis. Cancer Epidemiol Prev Biomark.

[48] Ding J, Warren R, Warsi I, Day N, Thompson D, Brady M (2008). Evaluating the effectiveness of using standard mammogram form to predict breast cancer risk: case-control study. Cancer Epidemiol Prev Biomark.

[49] Boyd NF, Connelly P, Byng J, Yaffe M, Draper H, Little L (1995). Plasma lipids, lipoproteins, and mammographic densities. Cancer Epidemiol Prev Biomark.

[50] Afifi AM, Saad AM, Al-Husseini MJ, Elmehrath AO, Northfelt DW, Sonbol MB (2020). Causes of death after breast cancer diagnosis: a US population-based analysis. Cancer.

